# Genetic diversity and population structure of an insect‐pollinated and bird‐dispersed dioecious tree *Magnolia kwangsiensis* in a fragmented karst forest landscape

**DOI:** 10.1002/ece3.70094

**Published:** 2024-07-31

**Authors:** Yanfang Lin, Yingying Xiang, Sujian Wei, Qiwei Zhang, Yanhua Liu, Zhiyong Zhang, Shaoqing Tang

**Affiliations:** ^1^ Key Laboratory of Ecology of Rare and Endangered Species and Environmental Protection, Ministry of Education Guangxi Normal University Guilin China; ^2^ Guangxi Key Laboratory of Landscape Resources Conservation and Sustainable Utilization in Lijiang River Basin Guangxi Normal University Guilin China; ^3^ Wuzhou No. 18 Middle School Wuzhou China

**Keywords:** dioecy, genetic diversity, karst forest, *Magnolia kwangsiensis*, population structure

## Abstract

This study combined population genetics and parentage analysis to obtain foundational data for the conservation of *Magnolia kwangsiensis*. *M*. *kwangsiensis* is a Class I tree species that occurs in two disjunct regions in a biodiversity hotspot in southwest China. We assessed the genetic diversity and structure of this species across its distribution range to support its conservation management. Genetic diversity and population structure of 529 individuals sampled from 14 populations were investigated using seven nuclear simple sequence repeat (nSSR) markers and three chloroplast DNA (cpDNA) fragments. Parentage analysis was used to evaluate the pollen and seed dispersal distances. The nSSR marker analysis revealed a high genetic diversity in *M*. *kwangsiensis*, with an average observed (*Ho*) and expected heterozygosities (*He*) of 0.726 and 0.687, respectively. The mean and maximum pollen and seed dispersal distances were 66.4 and 95.7 m and 535.4 and 553.8 m, respectively. Our data revealed two distinct genetic groups, consistent with the disjunct geographical distribution of the *M*. *kwangsiensis* populations. Both pollen and seed dispersal movements help maintain genetic connectivity among *M*. *kwangsiensis* populations, contributing to high levels of genetic diversity. Both genetically differentiated groups corresponding to the two disjunct regions should be recognized as separate conservation units.

## Graphical abstract

This study combined population genetics and parentage analysis to generate information for the conservation of *Magnolia kwangsiensis*. Two significant outcomes of the analysis are: (a) both pollen and seed dispersal movements maintain genetic connectivity between populations, contributing to high levels of genetic diversity in *M*. *kwangsiensis*; (b) both genetically differentiated groups corresponding to the two disjunct regions should be recognized as separate conservation units.

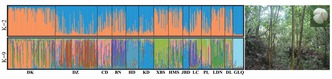

## 1 INTRODUCTION

The Karst Area in Southern China (KASC) harbors important reservoirs or “arks” of tropical and subtropical biodiversity, characterized by high levels of species diversity, endemism, and endangered plants (Clements et al., 2006; Yang et al., 2021). In a typical karst ecosystem, soil nutrients and moisture exhibit heterogeneity along topographic gradients, resulting in several karst microhabitats (Shen et al., 2019). The emergence of these karst ecological niches leads to the speciation and diversification of the karst flora (Clements et al., 2006). Plants that grow on limestone karst must adapt to its shallow soil, high Ca^2+^ and Mg^2+^ levels, and low water‐holding capacity, among other harsh challenges (Geekiyanage et al., 2019; Hao et al., 2015; Nie et al., 2011; Zhang et al., 2011). Extreme substrates, such as limestone, are often highly fragmented, restricting the distribution of many plant species to specialized substrates that tend to occur in spatially isolated patches, leading to small population sizes (Corlett & Tomlinson, 2020; Fan et al., 2017; Laanisto et al., 2012; Tamme et al., 2010). Moreover, human disturbance and climate change are adversely impacting this fragile ecosystem (Liu et al., 2023; Seidl et al., 2017). Thus, the endemic karst plant species are at risk of extinction due to several biotic and abiotic stressors (Duan et al., 2021; Li et al., 2013).

The fragmentation of plant populations, especially those of rare and endemic endangered species, might lead to inbreeding depression, fixation of deleterious mutations, and loss of adaptive genotypes (Aguilar‐Aguilar et al., 2023; Isagi et al., 2007; Martín‐Hernanz et al., 2019). Furthermore, long‐term fragmentation of plant populations is predicted to increase the chances of population differentiation due to genetic drift (Aguilar et al., 2008; Schultz & Scofield, 2009). The enhanced isolation of fragmented populations can affect pollen and seed dispersals, altering the patterns of gene flow and, thereby, impacting the genetic diversity among these populations. Previous studies have shown that strong geographical isolation by fragmentation can reduce pollen and seed migration and increase inbreeding (Gong et al., 2010); however, some studies have reported contrasting results (Gong & Gong, 2016; Wang et al., 2010; White et al., 2002). For instance, a previous study showed that the fragmented populations of the dioecious karst tree *Eurycorymbus cavaleriei*, a species visited by a broad spectrum of insect pollinators, can maintain genetic connectivity via long‐distance pollen gene flow (Wang et al., 2010). Differential patterns of pollen flow within fragmented populations are usually attributed to the composition and/or abundance of their pollinators and the foraging behavior of animals (Wang et al., 2010). Moreover, genetic connectivity among plant populations in natural landscapes is also determined by the distance and patterns of dispersed seeds (Ozawa et al., 2013). These studies suggested that different plant species exhibit varying responses to a fragmented landscape.

*Magnolia kwangsiensis* Figlar & Noot [*Woonyoungia septentrionalis* (Dandy) Y. W. Law] is a dioecious tree endemic to the limestone karst of China (Figlar & Nooteboom, 2004; Wu et al., 2008; Yang, Guo & Zailiu, 2018). This species currently occurs in two disjunct regions. One of these regions is located at the border of Guangxi and Guizhou, where populations are predominantly distributed in the Mulun National Nature Reserve and Maolan National Nature Reserve. This region comprises 72 fragmented distribution sites with a total of 5162 *M*. *kwangsiensis* individuals, with 29 sites harboring <5 individuals (Ling et al., 2021; Liu et al., 2020). The other disjunct region is in southeastern Yunnan (Yang, Guo & Zailiu, 2018). Due to its restricted geographic distribution, high habitat specificity, and slow natural regeneration (Fu et al., 2009, 2012; Ling et al., 2021), *M*. *kwangsiensis* has been listed in the Class I National Key Protected Wild Plant List of China (https://www.forestry.gov.cn/). Previously, a genetic structure analysis of five *M*. *kwangsiensis* populations, based on amplified fragment length polymorphism (AFLP), revealed significant genetic differentiations among the populations (Zhao et al., 2010). The dioecious nature of *M*. *kwangsiensis*, together with its pollination strategy (insect‐transmitted) and bird seed‐dispersal (Lai, Pan et al., 2007; Wang et al., 2019), can facilitate gene flow among the plant populations. Thus, a comprehensive understanding of the levels and patterns of genetic diversity in *M*. *kwangsiensis* across its entire distribution range is warranted.

In the present study, we analyzed the genetic diversity and population structure of *M*. *kwangsiensis* using seven nuclear simple sequence repeat (nSSR) markers and three chloroplast DNA (cpDNA) fragments. The study aimed to (i) characterize the level of genetic diversity in *M*. *kwangsiensis*, (ii) measure the pollen and seed gene flow distances and the patterns of genetic variability within *M*. *kwangsiensis* populations across a fragmented karst forest landscape, and (iii) discuss possible implications of these population genetic data for the management and conservation of *M*. *kwangsiensis*.

## 2 MATERIALS AND METHODS

### 2.1 Sample collection and DNA extraction

**FIGURE 1:**
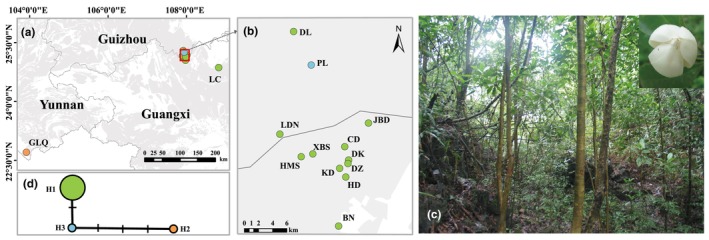
Geographical distribution of the *Magnolia kwangsiensis* populations (a, b). Photographs of the habitat and male flower (inset) of *M*. *kwangsiensis* (c). The network of *M*. *kwangsiensis* haplotypes based on chloroplast DNA (cpDNA) (d). The gray‐shaded areas on the map are karst landforms. The remaining colors represent the haplotypes. This map was made based on the standard map No. GS (2019) 1822 downloaded from the standard map service website of the Ministry of Natural Resources of the People's Republic of China. The base map is open‐access and has not been modified. The geological data of the southwestern karst region was obtained from the National Earth System Science Data Center, National Science & Technology Infrastructure of China.

We collected a total of 592 samples from 14 *M*. *kwangsiensis* populations in 2011, with 20–119 individuals per population. Of these populations, nine were located in the Mulun National Nature Reserve, Huanjiang County, Guangxi Province; three in the Maolan National Nature Reserve, Lipo County, Guizhou Province; one in Luocheng County, Guangxi Province; and one in the Gulinjing Provincial Nature Reserve, Maguan County, Yunnan Province. The populations in the Guangxi and Guizhou Provinces were defined as the eastern populations, and the population in Yunnan was defined as the western population. The details about these regions and their *M*. *kwangsiensis* populations are depicted in Table 1 and Figure 1a–c.

**TABLE 1:** Information for the *Magnolia kwangsiensis* sampling sites and their sample size.

	Locality	Population code	Longitude	Latitude	Elevation (m)	Sample size SSR (cpDNA)
Eastern	Dongkuai, Huanjiang, Guangxi	DK	107.98306°	25.13750°	637	119 (10)
	Dongzai, Huanjiang, Guangxi	DZ	107.98222°	25.13306°	696	106 (10)
	Changdong, Huanjiang, Guangxi	CD	107.97806°	25.15444°	650	37 (10)
	Bannan, Huanjiang, Guangxi	BN	107.97028°	25.05361°	535	35 (10)
	Hongdong, Huanjiang, Guangxi	HD	107.97944°	25.11583°	700	32 (10)
	Kudong, Huanjiang, Guangxi	KD	107.97167°	25.12694°	675	38 (10)
	Xiabaishan, Huanjiang, Guangxi	XBS	107.93750°	25.14528°	825	37 (10)
	Houmeishan, Huanjiang, Guangxi	HMS	107.92278°	25.14167°	701	36 (10)
	Jiabadong, Huanjiang, Guangxi	JBD	108.00833°	25.18444°	650	16 (10)
	Luocheng, Guangxi	LC	108.81861°	24.86556°	450	31 (10)
	Pailang, Maolan, Guizhou	PL	107.93556°	25.25806°	750	24 (10)
	Ladongning, Maolan, Guizhou	LDN	107.89556°	25.17028°	750	35 (10)
	Donglou, Maolan, Guizhou	DL	107.91306°	25.30056°	700	20 (10)
Western	Gulinjin, Maguan, Yunnan	GLQ	103.91139°	22.70861°	847	26 (10)
Total	–	–	–	–	–	592 (140)

**FIGURE 2:**
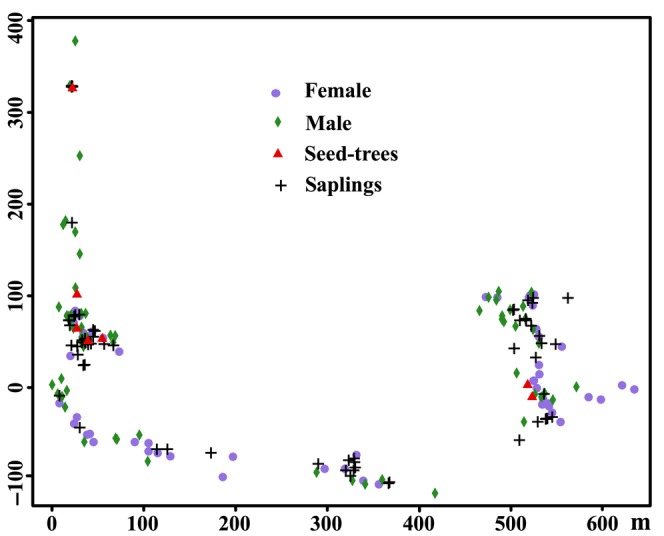
The distribution of *Magnolia kwangsiensis* individuals in the DK and DZ populations used for parentage analysis.

The position of all *M*. *kwangsiensis* individuals were located in DK and DZ and their junctions. A total of 332 plants were identified, including 67 male‐flowering, 63 female‐flowering, 65 saplings (diameter at breast height (DBH) < 2.5 cm), and 137 non‐flowering adult individuals that year (DBH > 2.5 cm). Leaves from all individuals and 329 mature seeds from seven seed‐trees were collected and used for the parentage analysis (Figure 2). The collected seeds were soaked in laundry detergent for 6–8 h, washed to remove the aril, stored in sterile sand, and germinated in a greenhouse (Lai, Huang et al., 2007). Their germination rate was 100%. Total genomic DNA was extracted from fresh leaves (dried using silica gel) using the cetyltrimethylammonium bromide (CTAB) method previously described by Doyle and Doyle (1987).

Seven nSSR markers (Ksep01, Ksep05, Ksep07, Ksep09, M6D3, stm0310, and stm0246) were selected for this study (see Appendix 1). M6D3, stm0310, and stm0246 and the remaining four markers were developed for *Magnolia obovata*, *Magnolia stellata*, and *M*. *kwangsiensis*, respectively (Isagi et al., 1999; Lin et al., 2011; Setsuko et al., 2005). The PCR mixture for each sample comprised 2 μL of 10× PCR buffer, 0.4 μL of MgCl_2_ (25 mM), 0.4 μL of dNTPs (10 mΜ), 0.2 μL of each primer (50 μM), 0.2 μL of rTaq polymerase (all these reagents were obtained from TaKaRa, China), 1 μL of sample DNA (20–30 ng), and 15.6 μL of ddH_2_O (total volume: 20 μL). The PCR protocol was as follows: An initial denaturation step at 94°C for 10 min, followed by 30 cycles of denaturation at 94°C for 45 s, annealing at the appropriate temperature for 45 s for each primer pair, and an extension at 72°C for 45 s. A final elongation step was performed for each reaction at 72°C for 10 min. Allele and genotype frequencies for each locus were obtained from the data reads using a 6% polyacrylamide denaturing gel electrophoresis with a 10 bp DNA ladder (Invitrogen) as the reference. The bands were visualized via silver staining.

### 2.2 Amplifying and sequencing the intergenic regions in chloroplast DNA

We amplified and sequenced cpDNA fragments from five individuals in each population. A total of 15 primer pairs were used (see Appendix 1). Among these primers, those with polymorphic loci were used to amplify and sequence the cpDNA from five more individuals from each population. The primer pairs were derived from the large and small single‐copy (LSC and SSC) regions previously described by Shaw et al. (2005, 2007). PCR mixture for each sample comprised 5 μL of 10× PCR buffer (containing Mg^2+^), 4 μL of dNTPs (2.5 mM), 0.5 μL of each forward and reverse primers (50 μM), 1 U rTaq polymerase (all reagents from TaKaRa), 1 μL of DNA solution (20–30 ng), and 38.5 μL of ddH_2_O (total volume: 50 μL). The PCR amplifications were performed in a thermocycler using the same protocol used for nSSR amplification described in Section 2.1. The PCR products were directly purified and sequenced using a 3730XL DNA Analyzer (Applied Biosystems).

### 2.3 Parentage analysis

Parentage analysis was conducted using the CERVUS v3.0 software based on the exclusion and maximum likelihood methods (Corlett & Tomlinson, 2020) to estimate pollen and seed distribution rates for DK and DZ, within a 500 m × 600 m plot (Jones et al., 2010). In the exclusion method, zero mismatches were accepted. For the likelihood method, five parameters were set as follows: Minimum number of matching loci: 7, error rate: 0.01, number of candidate parents: 130 (including 63 mother and 67 father trees), the proportion of candidate parents sampled: 0.95, and proportion of loci typed: 0.99 (Garcia et al., 2005). The critical log odds ratio (LOD) scores with 95% (strict) and 80% (relaxed) levels of confidence were calculated based on 10,000 simulations. The most likely father or mother was assigned when the confidence level was >80%, and the pair loci mismatching was 0.

*Magnolia kwangsiensis* is a dioecious tree (Wu et al., 2008). Given that the mother tree of the harvested seeds is known, the effective pollen dispersal distance was determined by calculating the distance between the two parents when the father of the seeds was identified using parentage analysis of the plants with male flowers in the current year. Similarly, when the mother tree of the saplings was identified through parentage analysis of the plants with female flowers in the current year, the effective seed dispersal distance was determined by calculating the distance of the parent from the saplings.

### 2.4 SSR analysis

The Hardy–Weinberg equilibrium and linkage disequilibrium (LD) tests were conducted using the GENEPOP v4.1 software (Raymond & Rousset, 1995). The exact level test was performed using a Markov‐chain random program with the following parameters: 10,000 dememorizations, 5000 batches, and 5000 iterations per batch.

Diversity measures, such as number of alleles per locus (*Na*), effective number of alleles (*Ne*), observed heterozygosity (*Ho*), expected heterozygosity (*He*), and the genetic differentiation coefficient (*F*_ST_), were calculated using the GenAIEx v6.5 software (Peakall & Smouse, 2012). A principal coordinates analysis (PCoA) was undertaken using GenAlEx to derive the patterns of genetic differentiation based on pairwise *F*_ST_ values among the populations. Gene flow (*Nm*), based on *F*_ST_ values, was calculated using the formula *Nm* = [(1 − *F*_ST_)/4*F*_ST_] based on the migration‐drift equilibrium hypothesis (Wright, 1970). Next, we performed a Mantel test using GenAIEx v6.5 to assess whether genetic differentiation was primarily impacted by isolation by distance (IBD). Analysis of Molecular Variance (AMOVA) was conducted using the ARLEQUIN v.3.5 software to compare differences among the groups, among the samples within the same group, and among all samples (Excoffier et al., 2005). For this analysis, two regional groups, comprising the eastern (DK, DZ, CD, BN, HD, KD, XBS, HMS, JBD, LC, PL, LDN, and DL) and western (GLQ) populations, were compared. To assess the significance of the differences, 10,000 permutations were used for the AMOVA calculations.

We used STRUCTURE v2.3.4 software to analyze the population structure of *M*. *kwangsiensis* (Pritchard et al., 2000). We conducted 10 independent runs, with a burn‐in period of 10^5^ steps, followed by 10^6^ iterations, to estimate the ideal population size (*K*) ranging from 1 to 14. The admixture model was specified for each run. To identify the most probable *K* value, we used the delta *K* method as described previously (Evanno et al., 2005). The ideal number of population clusters was determined by the highest *K* value estimated by the STRUCTURE HARVESTER program (Earl & Vonholdt, 2011). The final merged results were obtained using the CLUMPP v1.1 (Jakobsson & Rosenberg, 2007) and DISTRUCT v1.1 programs (Rosenberg, 2003). A phylogenetic tree of the 14 plant populations was built using the unweighted pair group method with arithmetic mean (UPGMA), based on Nei's (1983) genetic distance, using the Populations v1.2.32 software (http://bioinformatics.org/~tryphon/populations/).

Based on the nSSR data, BARRIER v2.2 (Manni et al., 2004) was used to identify potential barriers to gene flow among the plant populations. A Voronoï tessellation map was created using Delaunay triangulation and the geographical coordinates of the 14 sample sites. Each population was contained in a polygon with edges adjacent to the neighboring sampling points. Then, Monmonier's (1973) maximum difference algorithm was used to build potential barriers among the neighboring populations through the subdivision map and a matrix of *D* genetic distances (Jost, 2008). The analysis was repeated on 500 resampled bootstrap *D* matrices in the R package “diversity” v1.9.90 (Keenan et al., 2013) to assess the robustness of the calculated barriers.

We used the BOTTLENECK software (Piry et al., 1999) to detect the possible occurrence of a bottleneck in each population. For this analysis, the Wilcoxon signed rank test and the two‐phase model (TPM) were used. The default settings for the TPM included 30% and 70% variations from the infinite alleles model (Newton et al., 2008) and the strict stepwise mutation model (SMM), respectively.

### 2.5 cpDNA sequence analysis

All cpDNA sequences were assembled using SeqMan (Clewley, 1995) and aligned using the CLUSTAL X tool (Thompson et al., 1997). We obtained three polymorphic fragments from the 15 primer pairs. These fragments were further amplified and used for the haplotype analysis. The number of haplotypes (h) and polymorphic sites was calculated using the DNAsp v5.10 software (Librado & Rozas, 2009). The network was constructed using the median‐joining method (Bandelt et al., 1999) in the Network v5.0 software (Xu et al., 2020) to study the genealogical relationships among all the haplotypes. The geographical distribution of the populations and haplotypes was then mapped using ArcMap GIS v10.5 (ESRI) (Ueno et al., 2005). All the generated cpDNA sequences have been deposited in GenBank under accession numbers OP909724–OP909732, OQ848601–OQ848612 (Sayers et al., 2022).

A phylogenetic tree of the cpDNA haplotypes was constructed using the ML method in IQ‐TREE v1.6.12 (Nguyen et al., 2015). The chloroplast genome sequences of *Magnolia sinica* (NC 023241) and *Magnolia lotungensis* (NC 062929), downloaded from the GenBank public sequence database, were used as outgroups after removing redundant fragments (Sayers et al., 2022). The TIM + F + I model was chosen as the most appropriate substitution model according to the lowest Akaike Information Criterion (AIC) described in IQ‐TREE ModelFinder (Kalyaanamoorthy et al., 2017). The accuracy of the branching models was determined using 1000 bootstrap replications.

Divergence times for the three polymorphic fragments from the 15 primer pairs were estimated using the BEAST v1.6.1 software under a strict clock model (Drummond & Rambaut, 2007). Evolutionary rates of 1 × 10^−9^ substitutions/site/year and 3 × 10^−9^ substitutions/site/year were used based on the evolutionary rate of angiosperm cpDNA (Drummond & Rambaut, 2007). We used the general time‐reversible (GTR) substitution model with a gamma distribution and four rate categories. The performance of different Bayesian tree priors was determined according to Yule (yule.birthRate = uniform prior [0.0, ∞]). Two independent analyses (with different evolutionary rates) were run in BEAST for 100,000,000 generations of Markov chain Monte Carlo (MCMC). Finally, the Tracer 1.7 program was used to evaluate the convergence of the chain (Rambaut et al., 2018). The top 10% of Bayesian trees were filtered using the TreeAnnotator tool (in BEAST v1.6.1).

## 3 RESULTS

### 3.1 Genetic diversity based on nSSR markers

Diversity estimates for each locus were generalized (see Appendix 2). The seven selected microsatellite loci yielded a total of 88 alleles (~12.6 per locus), with Ksep07 and stm0246 yielding the fewest (*n* = 6) and the most alleles (*n* = 23), respectively. The Hardy–Weinberg equilibrium analysis showed that only five of 98 locus‐population combinations deviated from the equilibrium after the Markov‐chain random correction (*p* < .01). LD testing indicated significant linkages in 41 out of 294 locus pairs; however, the linked locus pairs were found in only four populations. These findings suggested the seven microsatellite markers were relatively independent in their generational transmission. Hence, they could provide a more realistic reflection of the genetic characteristics of *M*. *kwangsiensis*.

As shown in Table 2, *M*. *kwangsiensis* exhibited a high level of genetic diversity (*Na* = 6.663, *Ne* = 3.542, *Ho* = 0.726, and *He* = 0.687). Among the 14 populations, the BN and the DZ populations exhibited the lowest and highest levels of genetic diversities, respectively (*Na* = 5.143 and 8.571, *Ne* = 2.734 and 3.677, *Ho* = 0.522 and 0.748, and *He* = 0.568 and 0.701, respectively).

**TABLE 2:** Genetic variability of seven nuclear simple sequence repeat markers and the haplotypes of three chloroplast DNA (cpDNA) sequences, and results for the Wilcoxon test and the mode‐shift.

Pop.	Haplotypes (number)	SSR				Wilcoxon test	Mode‐shift
		Na	Ne	Ho	He		
DK	H1 (10)	8.571	3.677	0.748	0.701	0.766	L‐shaped
DZ	H1 (10)	8.571	4.205	0.805	0.752	0.148	L‐shaped
CD	H1 (10)	7.286	3.968	0.788	0.729	0.148	L‐shaped
BN	H1 (10)	5.143	2.734	0.522	0.568	0.406	L‐shaped
HD	H1 (10)	6.714	3.807	0.817	0.723	0.055	L‐shaped
KD	H1 (10)	6.143	3.281	0.718	0.689	0.148	L‐shaped
XBS	H1 (10)	6.429	3.907	0.676	0.684	0.406	L‐shaped
HMS	H1 (10)	6.857	3.631	0.675	0.699	0.234	L‐shaped
JBD	H1 (10)	5.714	3.536	0.821	0.696	0.027*	L‐shaped
LC	H1 (10)	6.143	3.185	0.664	0.649	0.148	L‐shaped
PL	H3 (10)	6.429	3.679	0.756	0.700	0.289	L‐shaped
LDN	H1 (10)	7.429	3.052	0.665	0.662	0.973	L‐shaped
DL	H1 (10)	5.429	3.597	0.779	0.697	0.148	L‐shaped
GLQ	H2 (10)	6.429	3.328	0.731	0.667	0.188	L‐shaped
Mean	–	6.663	3.542	0.726	0.687	–	–

*Note*: Haplotypes (number), haplotype types and number of cpDNA sequences.

Abbreviations: *He*, expected heterozygosity; *Ho*, observed heterozygosity; *Na*, number of mean alleles; *Ne*, number of effective alleles.

* *p* < .05.

### 3.2 Genetic structure deduction based on nSSR data

The *F*_ST_ and *Nm* values are shown in Table 3. The pairwise *F*_ST_ values ranged from 0.022 (DK and HMS) to 0.176 (GLQ and BN). However, the pairwise *F*_ST_ values between GLQ and eastern populations ranged from 0.086 (DZ) to 0.176 (BN). This range of values exceeded the *F*_ST_ values within the eastern populations (except for BN and PL). These results revealed a high genetic differentiation between GLQ and the eastern populations. The pairwise *Nm* values ranged from 2.221 (PL and LDN) to 11.230 (DK and HMS), indicating a high degree of gene flow in the eastern populations. AMOVA demonstrated that 11.46%, 8.29%, and 80.24% of the genetic variation could be attributed to intergroup differences, differences among the populations within the same group, and differences among all the populations, respectively (Table 4).

**TABLE 3:** Pairwise *Magnolia kwangsiensis* populations matrix of the genetic differentiation coefficient (*F*_ST_) (above the diagonal) and gene flow (*Nm*) (below the diagonal).

	DK	DZ	CD	BN	HD	KD	XBS	HMS	JBD	LC	PL	LDN	DL	GLQ
DK	–	0.043	0.040	0.052	0.059	0.051	0.052	0.022	0.065	0.058	0.078	0.032	0.045	0.126
DZ	5.603	–	0.029	0.064	0.046	0.048	0.060	0.039	0.059	0.045	0.055	0.060	0.043	0.086
CD	5.971	8.492	–	0.055	0.045	0.070	0.063	0.032	0.047	0.065	0.049	0.055	0.040	0.112
BN	4.533	3.677	4.258	–	0.092	0.085	0.087	0.046	0.087	0.055	0.102	0.068	0.089	0.176
HD	4.001	5.241	5.357	2.468	–	0.088	0.066	0.060	0.079	0.090	0.068	0.067	0.062	0.117
KD	4.661	4.932	3.315	2.675	2.579	–	0.070	0.053	0.072	0.077	0.075	0.075	0.075	0.121
XBS	4.577	3.914	3.703	2.631	3.51	3.336	–	0.045	0.090	0.057	0.051	0.076	0.043	0.120
HMS	11.23	6.116	7.508	5.208	3.888	4.477	5.362	–	0.061	0.046	0.078	0.030	0.041	0.118
JBD	3.6	4.006	5.048	2.612	2.915	3.203	2.525	3.853	–	0.090	0.082	0.095	0.077	0.125
LC	4.065	5.25	3.589	4.287	2.516	3.014	4.137	5.188	2.542	–	0.080	0.075	0.076	0.117
PL	2.969	4.256	4.815	2.198	3.401	3.062	4.685	2.968	2.787	2.892	–	0.101	0.060	0.128
LDN	7.508	3.941	4.257	3.411	3.491	3.097	3.023	8.072	2.384	3.103	2.221	–	0.054	0.136
DL	5.366	5.551	6.057	2.558	3.774	3.103	5.532	5.8	2.996	3.036	3.896	4.386	–	0.110
GLQ	1.73	2.655	1.99	1.171	1.888	1.816	1.84	1.871	1.745	1.881	1.709	1.585	2.016	–

**TABLE 4:** Analysis of molecular variance (AMOVA) based on simple sequence repeats of *Magnolia kwangsiensis*.

Source of variation	d.f.	Sum of squares	Variance components	Percentage variation (%)
Among groups	1	51.7	0.353 Va	11.46
Among populations within groups	12	285.4	0.255 Vb	8.29
Within populations	1170	2886.9	2.467 Vc	80.24
Total	591	3224.0	3.075	

*Note*: d.f. means degrees of freedom; variance components are significant (*p* < .05).

**FIGURE 3:**
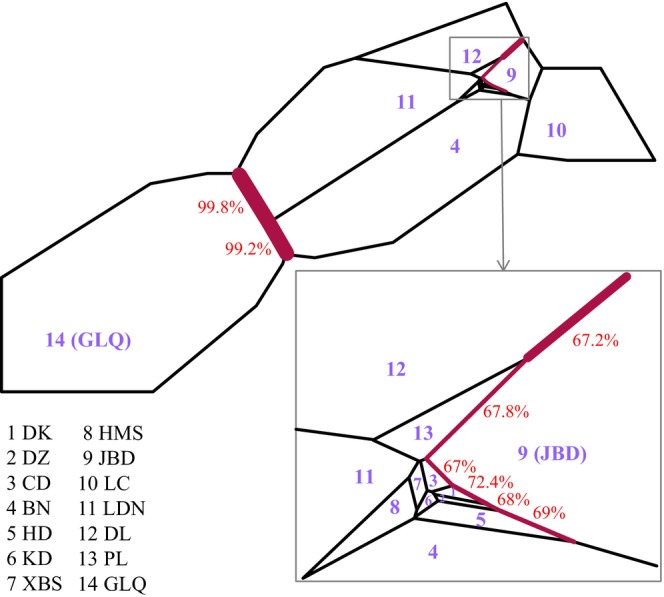
Delaunay triangulation network and Voronoï tessellation of the barrier analyses for *Magnolia kwangsiensis* and its close relatives. Black lines show Voronoï polygons, and red lines show inferred barriers. The thickness of the barriers (red lines) indicates their rank of importance, and the percentages indicate the degree of support. Only barriers with >60% support are shown.

The BARRIER analysis (Figure 3) revealed a strong gene flow barrier between the western and eastern populations with >99% support. Only JBD in the eastern population exhibited gene flow barriers with its adjacent populations (PL, CD, DK, DZ, HD, and DL), with statistical support ranging from 67% to 72%.

**FIGURE 4:**
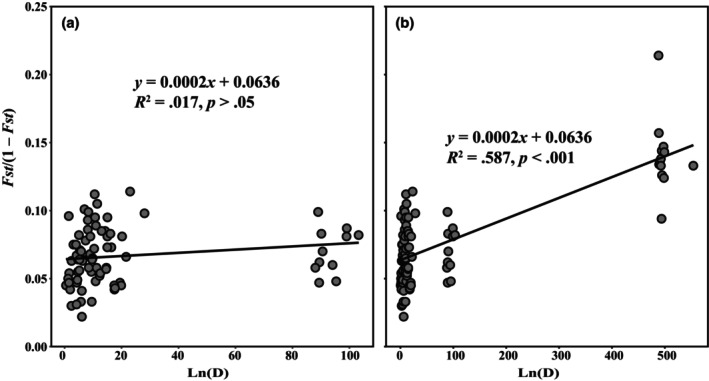
Correlation between genetic distance *F*_ST_/(1 − *F*_ST_) and geographic distance (GD) among 13 (a, excluding GLQ) and 14 (b, all of them) populations of *Magnolia kwangsiensis*.

The Mantel test results (Figure 4a,b) corroborated the significant effect of IBD in all populations (*R* ^2^ = .587, *p* < .001). However, the genetic and geographical distances of eastern populations only mildly correlated (*R* ^2^ = .017, *p* > .05).

**FIGURE 5:**
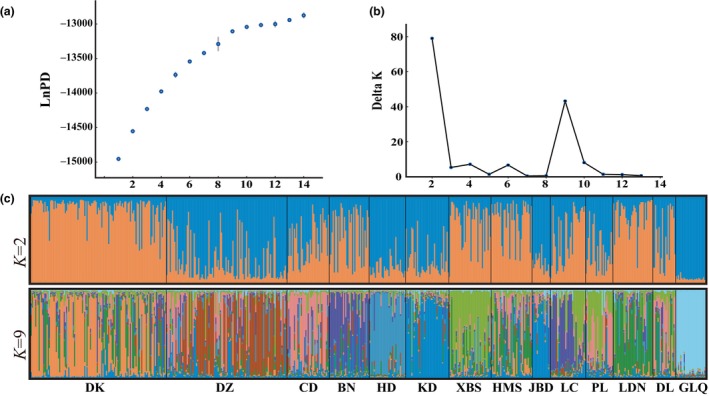
Scatterplot of the mean of the estimated ln probability of data (lnPD) versus the number of simulated clusters *K* (a), the relationship between *K* and Delta *K* (b), and the population structures with *K* = 2 and *K* = 9 (c) based on nuclear simple sequence repeat.

**FIGURE 6:**
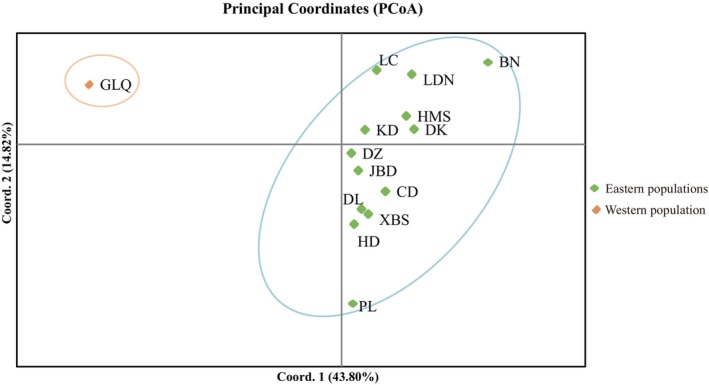
Principal coordinate analysis (PCoA) of all specimens from the 14 *Magnolia kwangsiensis* populations.

Analysis with STRUCTURE HARVESTER revealed that the number of delta *K* (Δ*K*) had clear peaks at 2 and 9 (Figure 5a,b). Nonetheless, based on the method previously described by Evanno et al. (2005), Δ*K* should be used together with ln probability of data (lnPD) to discern the optimal number of genetic populations. Thus, in the present study, the optimal number was 9. Furthermore, the Bayesian model suggested a clear separation between the western and eastern populations, with a mixed origin for the eastern populations (Figure 5c). Similar results were observed via the PcoA based on *F*_ST_ values and the UPGMA tree based on *Nei*'s genetic distance (Figure 6 and Appendix 4). These results indicated that the eastern populations did not exhibit a clear geographic genealogical structure, and the western and eastern populations exhibited a high genetic differentiation.

Bottleneck analyses showed that, based on TPM conditions, the allele distribution of each population exhibited a normal L‐shaped distribution, which was consistent with the mutation‐drift equilibrium. The Wilcoxon test revealed a significant heterozygosity in JBD (*p* < .05), indicating its deviation from the mutation‐drift equilibrium. However, none of the other 13 populations exhibited a recent genetic bottleneck (Table 2).

### 3.3 Parentage analysis

Based on the offspring‐mother genotype comparison, parentage analysis of the seven microsatellite loci yielded a total of 63 alleles (~9 alleles per locus). The fewest (*n* = 4) and the most (*n* = 15) alleles were detected for Ksep07 and stm0246, respectively. The abundance of genotypes enabled a thorough genetic analysis of the relationship between conspecific individuals. The analysis of genotyping errors revealed that the error rates between seeds and mother trees ranged from 0 (at locus Ksep07) to 0.035 (at locus stm0246), with all loci less than 5% (i.e., 0.05). Based on these results, seven corresponding primer pairs were selected for parentage analysis.

**FIGURE 7:**
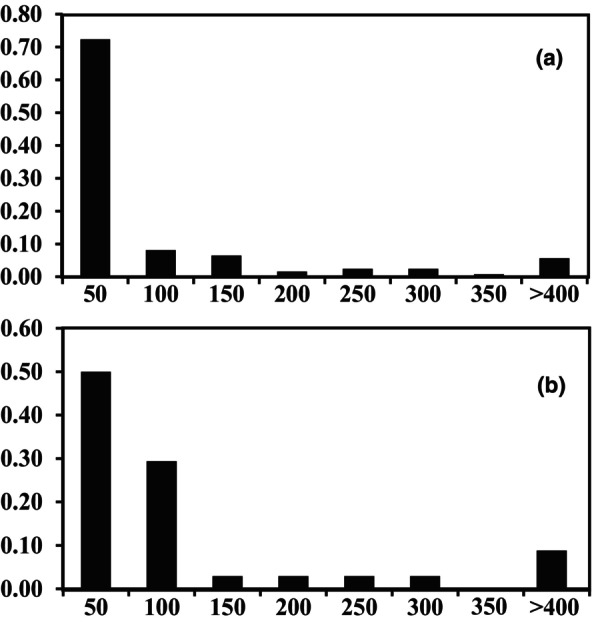
Distance versus frequency histograms of pollen (a) and seed (b) dispersal distance for *Magnolia kwangsiensis*.

Further parentage analysis helped identify the male parent of 132 seeds (40.12%) and the female parent of 34 saplings (52.3%; refer to Figure 7a,b and Appendix 3 for details). The pollen dispersal distance ranged from 2.2 to 535.4 m (average distance: 66.4 m). For seven out of 132 seeds (5.3%), pollen flow was identified between DK and DZ. The seed dispersal distance ranged from 0.2 to 553.8 m, averaging 95.7 m. For three out of 34 saplings (8.8%), seed dispersal was identified between DK and DZ. However, the female parents of the remaining specimens [*n* = 31/65 (47.7%)] could not be identified. It is possible that these female parents were present outside the study area or were among the 132 non‐flowering individuals.

### 3.4 Haplotypes based on cpDNA sequences

After gene tandem and alignment, the fragments amplified using the 15 primer pairs (see Appendix 1) had a total length of 14,703 bp. Only three primer pairs with polymorphisms—rpoB‐trnC, ndhF‐rpl32, and rpl32‐trnL—were selected for the haplotype analysis. After sequence splicing and artificial correction, we obtained 3663 bp long cpDNA sequences from 140 individuals. Three polymorphic loci were detected, and three chloroplast haplotypes were uncovered (H1–H3, see Appendix 5). The different haplotypes and their network and geographical distribution are presented in Table 2 and Figure 1a,b,d. Evidently, haplotype H1 was the most abundant and widely distributed. It was found in 12 populations: DK, DZ, CD, BN, HD, KD, XBS, HMS, JBD, LC, LDN, and DL. In stark contrast, haplotypes H2 and H3 were unique to the GLQ and PL populations, respectively (Table 2). In the haplotype network, H2 was separated from H3 and H1 by three and four mutational steps, respectively (Figure 1d).

### 3.5 Molecular dating by cpDNA sequences

**FIGURE 8:**
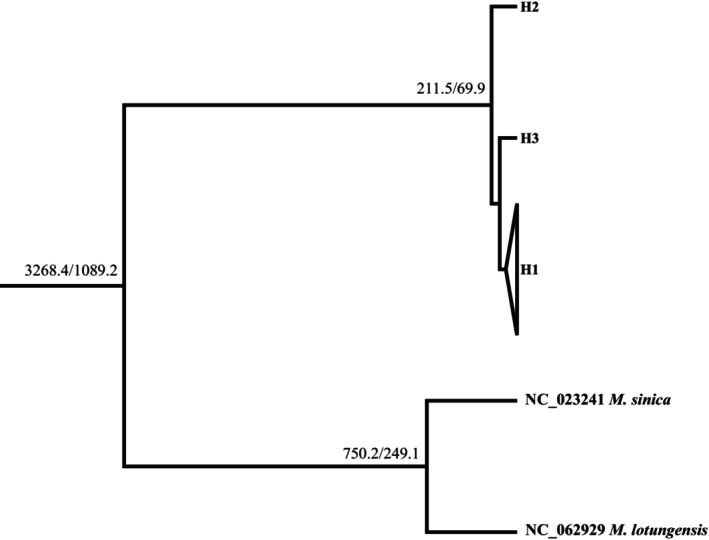
Maximum likelihood phylogenetic tree of the three chloroplast haplotypes (H1–H3) detected in *Magnolia kwangsiensis* using *M*. *sinica* and *M*. *lotungensis* as the outgroups. Each internal node is labeled with the posterior probabilities with rates of 1 × 10^−9^ substitutions/site/year and 3 × 10^−9^ substitutions/site/year. The scale represents thousands of years ago (ka).

The phylogenetic tree built using the ML method divided the three haplotypes into two genetic lineages (Figure 8). Further analysis revealed that the divergence time between the western (H2) and eastern populations spanned 211.5 ka (1 × 10^−9^ substitutions/site/year) to 69.9 ka (3 × 10^−9^ substitutions/site/year) before the present (BP) (Figure 8).

## 4 DISCUSSION

### 4.1 Genetic differentiation between two disjunct regions

The chloroplast haplotype tree, structure analysis, PCoA, and UPGMA tree analysis indicated that the 14 populations split into two differentiated groups, which was consistent with their disjunct geographical distribution (Figures 5c and 6 and Appendix 4). BARRIER analysis showed a strong gene flow barrier between the two differentiation groups (Figure 3). Additionally, AMOVA suggested that intergroup variation was higher (11.46%) than the variation among populations within the same group (8.29%) (Table 4). The divergence time between western (GLQ) and eastern populations was estimated to be ca. 221.5–69.9 ka (Figure 8), which seemed to coincide with the Last Glacial Period (LGP) (Yi et al., 2005). Therefore, we speculated that *M*. *kwangsiensis* persisted in both these regions during the glacial periods, resulting in the current disjunct distributions of its populations. Limestone mountains have been widely linked to refugia (Bátori et al., 2019). The refugia in the KASC evolved to support *Cycas* (Tao et al., 2021), *Primulina* (Ke et al., 2021), and *Heteroplexis* (Zhu et al., 2022). However, the complex topography of the karst landscape can buffer the extreme climatic conditions, allowing for the survival of existing species and the emergence of new lineages (Hewitt, 2000; Tzedakis et al., 2002).

### 4.2 Strong gene flow among the eastern populations

The *F*_ST_ values indicated a high level of gene flow among the eastern populations. The gene flow of *M*. *kwangsiensis* is achieved through pollen and seed transmissions. Among the overall pollen flow among the selected population, ~5.3% occurred between DK and DZ (>400 m). Furthermore, the longest pollen dispersal distance was 535.4 m. These findings indicated that long‐distance pollen dispersal could occur among the *M*. *kwangsiensis* populations. The pollen dispersal distances detected in the present study were consistent with those observed for the same genus *M*. *stellata* (Setsuko et al., 2007) and *M*. *obovata* (Isagi et al., 2004), with a corresponding maximum dispersal distance of 420 m and 500, respectively. Thrips are the most frequent and effective pollinators of *M*. *kwangsiensis* (Lai, Pan et al., 2007). Although thrips cannot fly over long distances (Denmark et al., 1996), they can spread farther with wind support (Lewis, 1991). Hence, thrip pollination can be viewed as an intermediate strategy between wind pollination and insect pollination (Chan & Appanah, 1980). These findings indicated that thrips might be dispersed by the wind over long distances while carrying *M*. *kwangsiensis* pollen on their bodies.

Pollen dispersal can contribute to gene exchange between close populations, but gene exchange among *M*. *kwangsiensis* populations over long distances is more likely mediated via seed dispersal. In the present study, we observed an average seed dispersal distance of 95.7 m, with the maximum dispersal distance detected between DK and DZ (553.8 m). Additionally, 8% of the overall seed dispersal occurred between DK and DZ (>400 m). Moreover, we could not trace the female parents of 47.7% of the dispersed seeds, indicating that these seeds might have dispersed from outside populations. *M*. *kwangsiensis* seeds are known to be dispersed by 14 frugivorous bird species, such as the chestnut bulbul (*Hemixos castanonotus*), striated yuhina (*Yuhina castaniceps*), and scarlet minivet (*Pericrocotus flammeus*) (Wang et al., 2019). In addition, frugivorous birds can facilitate long‐distance seed dispersal (Isagi et al., 2004; Mueller et al., 2014; Viana et al., 2016). Seed dispersal is the only way populations can exchange individuals or colonize new habitats. This ecological process is critical for the survival of plant species in patchy landscapes (Browne & Karubian, 2018). There are 72 *M*. *kwangsiensis* fragmented distribution sites in the eastern distribution region (Ling et al., 2021; Liu et al., 2020). Although most sites harbor only a few individuals, they can serve as stepstones for gene exchange. Long‐distance pollen and seed dispersal, combined with these stepstones for gene exchange, help maintain genetic connectivity among the fragmented *M*. *kwangsiensis* populations.

### 4.3 High genetic diversity in *M*. *Kwangsiensis*

Contrary to the general expectation from a spatially isolated rare species, *M*. *kwangsiensis* does not exhibit a reduced genetic diversity at either the population or the species level unlike a related Magnoliaceae species (Table 5) (Fan et al., 2020; Kikuchi & Osone, 2021; Sun et al., 2011; Tamaki et al., 2008, 2018; Wang et al., 2018; Xiao et al., 2023; Xiong et al., 2014; Yang, Yang & Li, 2018; Zhang et al., 2017; Zhao et al., 2012; Zhou et al., 2021; Deng et al., 2020). Obligate outcrossing plant species tend to be more genetically diverse (Hamrick & Godt, 1996; Jia et al., 2015; Muyle et al., 2020; Wang et al., 2010; Zong et al., 2015). Given that dioecious species are rare in the Magnoliaceae, our findings might be attributed to the dioecy of *M*. *kwangsiensis*, which might help its populations to avoid inbreeding and facilitate the involvement of many paternal individuals in the mating process. Moreover, our analysis did not reveal any recent bottleneck in *M*. *kwangsiensis*. Effective gene exchange via pollen and seed dispersal between isolated population patches helps to retain the genetic diversity of *M*. *kwangsiensis*.

**TABLE 5:** Comparison of genetic diversity between *Magnolia kwangsiensis* and other Magnoliaceae species.

Species	Na	Ho	He	*F*_ST_	Method	References
*Magnolia kwangsiensis*	12.6	0.726	0.687	0.125	SSR	This paper
*Magnolia stellata*	22.1	0.666	0.719	0.185	SSR	Tamaki et al. (2008)
*Magnolia sieboldii* subsp. *japonica*	1–7.2	0–0.6	0–0.664	0.636	SSR	Kikuchi and Osone (2021)
*Magnolia sieboldii* subsp. *sieboldii*	2.8–5.6	0.375–0.586	0.442–0.622	0.140	SSR	Kikuchi and Osone (2021)
*Magnolia patungensis*	–	0.349	0.307	0.340	SSR	Fan et al. (2020)
*Magnolia salicifolia*	27.8	–	0.782	0.133	SSR	Tamaki et al. (2018)
*Magnolia sinostellata*	–	0.21	0.52	0.236	EST‐SSR	Wang et al. (2019)
*Michelia maudiae*	8.2	0.457	–	0.171	SSR	Sun et al. (2011)
*Michelia coriacea*	4.091	0.412	0.505	0.090	SSR	Zhao et al. (2012)
*Michelia shiluensis*	14.375	0.686	0.718	0.139	SSR	Deng et al. (2020)
*Michelia yunnanensis*	4.329	0.554	0.537	0.058	SSR	Zhang et al. (2017)
*Michelia crassipes*	15.571	0.445	0.536	0.185	SSR	Xiao et al. (2023)
*Sinomanglietia glauca*	2.604	0.451	0.423	0.425	SSR	Xiong et al. (2014)
*Houpoea officinalis*	15.417	0.197	0.6	0.327	SSR	Yang et al. (2018)
*Liriodendron chinense*	2.707	0.262	0.399	0.302	SSR	Zhou et al. (2021)

Abbreviations: *F*_ST_, genetic differentiation coefficient; *He*, expected heterozygosity; *Ho*, observed heterozygosity; *Na*, number of mean alleles.

### 4.4 Conservation implications

Paternity analysis demonstrated that isolated patchy trees exhibit the potential for long‐distance pollen dispersal, a phenomenon also observed in other dioecious tree species in the KASC (Wang et al., 2014). Our results showed that pollen and seed dispersal can and does occur among *M*. *kwangsiensis* populations over large distances in highly fragmented karst forest landscapes. These phenomena further benefit from substantial intra‐ and inter‐patch pollinator movement and bird‐mediated dispersal because they enhance genetic connectivity across the fragmented landscape, constituting a functional network of gene flow and exchange. Nevertheless, two genetic groups of *M*. *kwangsiensis*, consistent with their disjunct distribution, were identified. Therefore, ensuring sufficient local tree densities and that inter‐fragment distances are maintained within a certain range could potentially reduce the negative genetic consequences of the fragmented karst habitat, even in species with the potential for long‐distance pollen‐ and seed‐mediated gene flow. Considering the current *M*. *kwangsiensis* population structure, its disjunct population groups should be treated as separate management units. Notably, LC lay outside the nature reserve, and JBD exhibited gene flow barriers against other populations. Therefore, LC and JBD deserve particular conservation attention.

## 5 CONCLUSIONS

Both pollen flow and seed dispersal helped maintain genetic connectivity among isolated *M*. *kwangsiensis* populations, resulting in its high genetic diversity across the karst landscape. The two genetically differentiated groups analyzed in this study were consistent with the two disjunct regions of the species. Our results provided a robust genetic basis for the conservation of *M*. *kwangsiensis*.

## AUTHOR CONTRIBUTIONS

**Shaoqing Tang:** Conceptualization (equal); funding acquisition (lead); project administration (lead); supervision (lead); validation (lead); visualization (lead); writing – review and editing (equal). **Zhiyong Zhang:** Conceptualization (equal); writing – review and editing (supporting). **Sujian Wei:** Conceptualization (equal); supervision (supporting); writing – review and editing (equal). **Yingying Xiang:** Conceptualization (equal); data curation (supporting); formal analysis (supporting); validation (supporting); visualization (supporting); writing – original draft (lead); writing – review and editing (supporting). **Yanfang Lin:** Conceptualization (equal); data curation (lead); formal analysis (lead); investigation (equal); methodology (lead); software (lead). **Yanhua Liu:** Data curation (supporting); investigation (equal). **Qiwei Zhang:** Data curation (supporting); formal analysis (supporting); investigation (equal); methodology (supporting); software (supporting).

## FUNDING INFORMATION

This study was supported by the Guangxi Natural Science Foundation (2010GXNSFA013069) and the Survey and Assessment of priority areas for terrestrial biodiversity conservation in Guangxi (2022–2023).

## CONFLICT OF INTEREST STATEMENT

The authors declare no conflict of interest.

## Data Availability

Voucher specimens were deposited in the Herbarium of Guangxi Institute of Botany (IBK) under the voucher numbers IBK00446213. The data that support the findings of this study are openly available in the NCBI. Nucleotide database at https://www.ncbi.nlm.nih.gov/nuccore/, under accession number: OP909724–OP909732, OQ848601–OQ848612.

## APPENDIX 1 PRIMERS USED TO AMPLIFY NUCLEAR SIMPLE SEQUENCE REPEAT AND CHLOROPLAST DNA

### 1.1

**Table:**

	Locus	Sequence (5′–3′)	Ta (°C)	Size (bp)	References
SSR	Ksep01	F: ACC CTA ACA GCA TTC ACT	55	116–154	Lin et al. (2011)
		R: GTC CCG GTG CGT AGA TAA			
	Ksep05	F: CCA ACT ACC ACA AAA AAA CAC	52	163–193	Lin et al. (2011)
		R: AAT CAC AAC AGG ACC CAA AAC			
	Ksep07	F: AGT GGG GTG ACA TGA GGT G	60	166–178	Lin et al. (2011)
		R: CAA AGG GGA GAT AGG GTG G			
	Ksep09	F: TCT ACA AAT ACT CCT CCA TCC	54	129–149	Lin et al. (2011)
		R: GGA CTA AGA TAC GGG TCT AAT			
	M6D3	F: ACA TGG ATA GTC GTT GGA TA	50	136–156	Isagi et al. (1999)
		R: ACC CCA CTG AAG ACA AAC AT			
	stm0246	F: AAG CAA AGC CTC CTA GGT C	58	173–221	Setsuko et al. (2005)
		R: TCT ACG CCT AAC AGG TCT GTC			
	stm0310	F: GCA CCG CTC TCG CTA TAA A	60	109–129	Setsuko et al. (2005)
		R: AAT CCG CGT CCG ACT ATA CC			
cpDNA	*rpoB–trnC*	rpoB: CKACAAAAYCCYTCRAATTG	50–57	1224	Shaw et al. (2005)
		trnC^GCA^R: CACCCRGATTYGAACTGGGG			
	*trnH–psbA*	trnH^GUG^: CGCGCATGGTGGATTCACAATCC	50–56	430	Shaw et al. (2005)
		psbA: GTTATGCATGAACGTAATGCTC			
	*psbA*–3'*trnK*–*matK*	matK8F: TCG ACT TTC TTG TGC TAG AAC TTT	50	762	Shaw et al. (2005)
		psbA5'R: AAC CAT CCA ATG TAA AGA CGG TTT			
	*rpS16*	rpS16F: AAA CGA TGT GGT ARA AAG CAA C	50–55	719	Shawoey et al. (2005)
		rpS16R: AAC ATC WAT TGC AAS GAT TCG ATA			
	*trnC–ycf6*	trnC^GCA^F: CCA GTT CRA ATC YGG GTG	50–55	932	Shaw et al. (2005)
		ycf6R: GCC CAA GCR AGA CTT ACT ATA TCC AT			
	*trnL*5'*–trnF*	trnL5'^UAA^F (TabC): CGA AAT CGG TAG ACG CTA CG	50	838	Shaw et al. (2005)
		trnF^GAA^ (TabF): ATT TGA ACT GGT GAC ACG AG			
	*trnT–trnL*	trnT^UGU^F (TabA): CAT TAC AAA TGC GAT GCT CT	50	1184	Shaw et al. (2005)
		3'trnL^UAA^R (TabD): GGG GAT AGA GGG ACT TGA AC			
	*rpL16*	rpL16F71: GCT ATG CTT AGT GTG TGA CTC GTT G	50	883	Shaw et al. (2005)
		rpL16R1516: CCC TTC ATT CTT CCT CTA TGT TG			
	3'*rps16*–5'*trnK*	rpS16x2F2: AAA GTG GGT TTT TAT GAT CC	45	641	Shaw et al. (2005)
		psbE: TAT CGA ATA CTG GTA ATA ATA TCA GC			
	*psaI–accD*	accD: AAT YGT ACC ACG TAA TCY TTT AAA	44	717	Shaw et al. (2005)
		psaI‐75R: AGA AGC CAT TGC AAT TGC CGG AAA			
	*ndhJ–trnF*	ndhJ: ATG CCY GAA AGT TGG ATA GG	50	1123	Shaw et al. (2005)
		TabE: GGT TCA AGT CCC TCT ATC CC			
	*psbD–trnT*	psbD: CTCCGTARCCAGTCATCCATA	47	1464	Shaw et al. (2005)
		trnT ^(GGU)^‐R: CCC TTT TAA CTC AGT GGT AG			
	*trnQ–*5'*rps16*	trnQ ^(UUG)^: GCG TGG CCA AGY GGT AAG GC	49	1768	Shaw et al. (2005)
		rpS16x1: GTT GCT TTY TAC CAC ATC GTT T			
	*ndhF–rpl32*	ndhF: GAA AGG TAT KAT CCA YGM ATA TT	55	1134	Shaw et al. (2005)
		rpL32‐R: CCA ATA TCC CTT YYT TTT CCA A			
	*rpl32–trnl*	rpL32‐F: CAG TTC CAA AAA AAC GTA CTT C	47	1304–1305	Shaw et al. (2005)
		trnL^(UAG)^: CTG CTT CCT AAG AGC AGC GT			

## APPENDIX 2 MICROSATELLITE MARKERS OF HETEROZYGOSITY AND *F* STATISTICS

### 2.1

**Table:**

Locus	*Na*	*Ho*	*He*	*F*_is_	*F*_st_
Ksep01	15	0.77	0.709	−0.085	0.139
Ksep05	13	0.764	0.701	−0.089	0.119
Ksep07	6	0.698	0.653	−0.068	0.063
Ksep09	11	0.685	0.627	−0.092	0.18
stm0310	9	0.755	0.696	−0.085	0.122
stm0246	23	0.701	0.781	0.102	0.108
M6D3	11	0.709	0.642	−0.105	0.153
Mean	12.6	0.726	0.687	−0.06	0.125

Abbreviations: *F*_is_, inbreeding coefficient; *F*_st_, genetic differentiation coefficient; *He*, expected heterozygosity; *Ho*, observed heterozygosity; *Na*, number of mean alleles.

## APPENDIX 3 THE RESULT OF PATERNITY ANALYSIS WITH THE EXCLUDE AND LIKELIHOOD METHODS

### 3.1

**Table:**

Offspring ID	Offspring ID	Candidate parents ID	Pair loci compared	Pair loci mismatching	Pair LOD score^a^
1	DK5	DK73	7	0	1.940^b^
2	DK13	DK306	7	0	4.410^b^
3	DK19	DK37	7	0	3.610^b^
4	DK25	DK52	7	0	3.550^b^
5	DK30	DK37	7	0	5.470^b^
6	DK44	DK37	7	0	5.970^b^
7	DK46	DK37	7	0	4.750^b^
8	DK66	DK85	7	0	4.250^b^
9	DK74	DK140	7	0	3.100^b^
10	DK78	DK27	7	0	3.720^b^
11	DK83	DK140	7	0	6.160^a^
12	DK87	DK311	7	0	0.950^b^
13	DK103	DK27	7	0	3.990^b^
14	DK107	DK115	7	0	3.100^b^
15	DK155	DK154	7	0	4.150^b^
16	DK157	DK154	7	0	1.530^b^
17	DK162	DK154	7	0	3.030^b^
18	DK300	DK179	7	0	3.370^b^
19	DK309	DK321	7	0	4.580^b^
20	DK319	DK339	7	0	3.230^b^
21	DK322	DZ249	7	0	6.120^b^
22	DK325	DK323	7	0	6.910^b^
23	DK342	DK328	7	0	7.120^b^
24	DK343	DK323	7	0	3.510^b^
25	DZ193	DZ233	7	0	2.410^b^
26	DZ195	DK154	7	0	4.930^b^
27	DZ220	DZ262	7	0	2.670^b^
28	DZ223	DK91	7	0	2.500^b^
29	DZ224	DZ262	7	0	3.100^b^
30	DZ236	DZ277	7	0	6.050^b^
31	DZ241	DZ233	7	0	7.540^b^
32	DZ269	DZ230	7	0	7.150^b^
33	DZ281	DZ249	7	0	6.560^b^
34	DZ405	DK54	7	0	5.040^b^
35	DK140‐4	DK72	7	0	3.406^b^
36	DK140‐13	DK72	7	0	6.506^b^
37	DK140‐14	DK41	7	0	6.062^b^
38	DK140‐16	DK8	7	0	5.210^b^
39	DK140‐18	DK41	7	0	5.905^b^
40	DK140‐19	DK14	7	0	4.330^a^
41	DK140‐21	DK149	7	0	4.656^b^
42	DK140‐22	DK141	7	0	6.497^b^
43	DK140‐23	DK41	7	0	6.687^b^
44	DK140‐25	DK139	7	0	5.594^b^
45	DK140‐28	DK50	7	0	4.726^a^
46	DK140‐29	DZ211	7	0	4.598^a^
47	DK140‐34	DK149	7	0	4.712^b^
48	DK140‐35	DK50	7	0	3.943^b^
49	DK140‐38	DK72	7	0	4.842^b^
50	DK140‐41	DK98	7	0	6.850^b^
51	DK140‐46	DK141	7	0	6.042^a^
52	DK32‐1	DK28	7	0	5.811^b^
53	DK32‐2	DK11	7	0	4.709^a^
54	DK32‐3	DK31	7	0	1.736^b^
55	DK32‐4	DK11	7	0	4.454^b^
56	DK32‐6	DK31	7	0	6.204^a^
57	DK32‐9	DK31	7	0	6.484^a^
58	DK32‐10	DK11	7	0	4.458^a^
59	DK32‐11	DK31	7	0	6.617^a^
60	DK32‐13	DK31	7	0	4.734^a^
61	DK32‐17	DK28	7	0	4.010^a^
62	DK32‐18	DK31	7	0	6.001^a^
63	DK32‐20	DK31	7	0	6.393^a^
64	DK32‐21	DK31	7	0	7.762^b^
65	DK32‐23	DK31	7	0	7.413^a^
66	DK32‐24	DK31	7	0	4.416^b^
67	DK32‐26	DK31	7	0	7.663^a^
68	DK32‐31	DK11	7	0	6.375^a^
69	DK32‐32	DK28	7	0	5.697^a^
70	DK32‐33	DK31	7	0	6.617^a^
71	DK32‐35	DK31	7	0	8.840^a^
72	DK32‐36	DK31	7	0	7.845^a^
73	DK32‐39	DK28	7	0	3.008^b^
74	DK32‐40	DK31	7	0	5.705^a^
75	DK32‐42	DK31	7	0	8.761^a^
76	DK32‐43	DK11	7	0	7.174^b^
77	DK32‐44	DK31	7	0	5.922^a^
78	DK32‐47	DK41	7	0	5.019^b^
79	DK32‐49	DK31	7	0	2.959^b^
80	DK32‐51	DZ287	7	0	6.057^a^
81	DK15‐3	DK14	7	0	2.630^b^
82	DK15‐7	DK17	7	0	2.400^b^
83	DK15‐9	DK97	7	0	6.740^b^
84	DK15‐11	DK11	7	0	3.787^b^
85	DK15‐12	DK14	7	0	6.869^a^
86	DK15‐14	DK14	7	0	4.995^b^
87	DK15‐15	DK11	7	0	3.982^b^
88	DK15‐17	DK11	7	0	5.045^b^
89	DK15‐22	DK14	7	0	6.306^b^
90	DK15‐23	DK14	7	0	4.250^b^
91	DK15‐26	DK317	7	0	4.799^b^
92	DK15‐30	DK121	7	0	6.488^a^
93	DK15‐34	DK11	7	0	4.866^a^
94	DK15‐35	DK14	7	0	5.236^b^
95	DK15‐38	DK11	7	0	6.386^a^
96	DK15‐39	DK14	7	0	4.360^b^
97	DK15‐42	DK11	7	0	4.356^b^
98	DK15‐43	DK7	7	0	4.579^b^
99	DK15‐48	DK14	7	0	6.353^a^
100	DK15‐49	DK11	7	0	4.233^b^
101	DK15‐50	DK11	7	0	5.043^b^
102	DK92‐1	DK317	7	0	3.129^b^
103	DK92‐4	DK94	7	0	4.740^b^
104	DK92‐5	DK94	7	0	4.927^b^
105	DK92‐8	DK14	7	0	3.360^b^
106	DK92‐9	DK94	7	0	6.621^b^
107	DK92‐12	DK341	7	0	4.525^b^
108	DK92‐18	DK181	7	0	6.357^a^
109	DK92‐20	DK139	7	0	4.270^b^
110	DK92‐24	DK94	7	0	5.600^b^
111	DK154‐4	DK94	7	0	6.223^b^
112	DK154‐12	DK163	7	0	3.902^b^
113	DK154‐14	DZ205	7	0	4.562^a^
114	DK154‐19	DK163	7	0	4.412^a^
115	DZ292‐2	DZ235	7	0	5.304^b^
116	DZ292‐4	DZ201	7	0	1.600^b^
117	DZ292‐7	DZ229	7	0	4.415^b^
118	DZ292‐8	DZ211	7	0	1.605^b^
119	DZ292‐10	DK344	7	0	4.072^a^
120	DZ292‐12	DK172	7	0	3.288^a^
121	DZ292‐22	DZ201	7	0	4.470^b^
122	DZ292‐24	DZ288	7	0	3.565^b^
123	DZ292‐26	DZ204	7	0	6.821^b^
124	DZ292‐29	DZ211	7	0	7.343^a^
125	DZ292‐31	DK136	7	0	4.012^b^
126	DZ292‐32	DZ288	7	0	3.633^a^
127	DZ292‐33	DZ288	7	0	2.917^b^
128	DZ292‐34	DZ187	7	0	5.614^b^
129	DZ292‐42	DZ201	7	0	0.898^b^
130	DZ292‐45	DZ273	7	0	3.670^b^
131	DZ292‐46	DZ287	7	0	6.309^a^
132	DZ292‐52	DZ270	7	0	3.507^a^
133	DZ292‐54	DZ211	7	0	3.237^b^
134	DZ292‐55	DZ270	7	0	5.904^b^
135	DZ292‐57	DZ273	7	0	6.885^b^
136	DZ292‐58	DZ252	7	0	3.478^b^
137	DZ292‐61	DZ287	7	0	2.637^b^
138	DZ290‐2	DZ211	7	0	3.918^a^
139	DZ290‐3	DZ235	7	0	8.966^a^
140	DZ290‐5	DZ270	7	0	6.295^a^
141	DZ290‐10	DK11	7	0	2.461^b^
142	DZ290‐11	DZ235	7	0	10.175^a^
143	DZ290‐13	DZ270	7	0	5.016^a^
144	DZ290‐14	DK50	7	0	3.966^a^
145	DZ290‐15	DZ270	7	0	3.967^a^
146	DZ290‐16	DZ288	7	0	6.822^a^
147	DZ290‐20	DK336	7	0	6.859^a^
148	DZ290‐25	DZ288	7	0	4.281^b^
149	DZ290‐26	DZ288	7	0	1.161^b^
150	DZ290‐27	DZ288	7	0	5.151^b^
151	DZ290‐28	DZ288	7	0	2.360^b^
152	DZ290‐30	DZ222	7	0	8.828^b^
153	DZ290‐33	DZ252	7	0	2.164^b^
154	DZ290‐35	DZ288	7	0	2.594^b^
155	DZ290‐37	DZ288	7	0	6.345^a^
156	DZ290‐40	DZ288	7	0	1.376^b^
157	DZ290‐41	DZ288	7	0	6.096^a^
158	DZ290‐42	DZ288	7	0	2.248^b^
159	DZ290‐49	DK336	7	0	1.456^b^
160	DZ290‐53	DZ187	7	0	6.576^a^
161	DZ290‐57	DZ288	7	0	5.840^b^
162	DZ290‐58	DZ211	7	0	1.978^b^
163	DZ290‐63	DK336	7	0	1.196^b^
164	DZ290‐65	DZ288	7	0	4.620^b^
165	DZ290‐68	DK344	7	0	7.003^b^
166	DZ290‐69	DZ287	7	0	6.057^a^

*Note*: Information about the female parents of 34 saplings is shown on the white background; Information about the male parents of 132 seeds is shown on the gray background.

^a^ 95% level of confidence.

^b^ 80% level of confidence.

## APPENDIX 5 MUTATION SITES OF *MAGNOLIA KWANGSIENSIS* IN CHLOROPLAST DNA HAPLOTYPE

### 5.1

**Table:**

Haplotype	Polymorphic site			
	208	1803	3386	1165
H1	G	A	T	b
H2	C	C	C	a
H3	G	A	C	b

^a^ Missing fragment.

^b^ ATACC.
